# Effects of Fermented *Artemisia annua* L. and *Salicornia herbacea* L. on Inhibition of Obesity In Vitro and In Mice

**DOI:** 10.3390/nu15092022

**Published:** 2023-04-22

**Authors:** Jeong-Yeon On, Su-Hyun Kim, Jeong-Mee Kim, Sungkwon Park, Ki-Hyun Kim, Choong-Hwan Lee, Soo-Ki Kim

**Affiliations:** 1 Department of Animal Science and Technology, Konkuk University, 120 Neungdong-ro, Gwangjin-gu, Seoul 05029, Republic of Korea; on7701@naver.com; 2 Department of Bioscience and Biotechnology, Konkuk University, Seoul 05029, Republic of Korea; 3 Institute of Animal Resource Center, Konkuk University, Seoul 05029, Republic of Korea; 4 Department of Food Science and Biotechnology, Sejong University, Seoul 05006, Republic of Korea; 5 Animal Welfare Research Team, National Institute of Animal Science, RDA, Wanju 55365, Republic of Korea; 6 Research Institute for Bioactive-Metabolome Network, Konkuk University, Seoul 05029, Republic of Korea

**Keywords:** annual wormwood, glasswort, metabolites, fermentation, anti-obesity, mouse

## Abstract

Plant extracts including secondary metabolites have anti-inflammatory and anti-obesity activities. This study was conducted to investigate the anti-obesity properties of fermented *Artemisia annua* (AW) and *Salicornia herbacea* (GW) in vitro and in mice. The metabolite profiling of AW and GW extracts was performed using UHPLC−LTQ−Orbitrap–MS/MS, and gene expression was analyzed using real-time PCR for adipocyte difference factors. The anti-obesity effects in mice were measured using serum AST, ALT, glucose, TG, and cholesterol levels. Metabolites of the plant extracts after fermentation showed distinct differences with increasing anti-obesity active substances. The efficacy of inhibitory differentiation adipogenesis of 3T3-L1 adipocytes was better for GW than AW in a concentration-dependent manner. RT-PCR showed that the GW extract significantly reduced the expression of genes involved in adipocyte differentiation and fat accumulation (C/EBPα, PPARγ, and Fas). In C57BL/6 mice fed the HFD, the group supplemented with AW and GW showed reduced liver weight, NAS value, and fatty liver by suppressing liver fat accumulation. The GW group significantly reduced ALT, blood glucose, TG, total cholesterol, and LDL-cholesterol. This study displayed significant metabolite changes through biotransformation in vitro and the increasing anti-obesity effects of GW and AW in mice. GW may be applicable as functional additives for the prevention and treatment of obesity.

## 1. Introduction

Obesity, which is considered a major public health problem, ranks as the fifth leading cause of death worldwide and has tripled in the last 40 years [1]. The World Obesity Atlas published in 2022 predicts that one billion people worldwide will live with obesity by 2030 [2]. The main cause of obesity is the increase in body fat storage efficiency because of a high-fat diet and the accumulation of fat due to adipogenesis by the differentiation of adipocytes [3]. Increased fat accumulation accompanies various diseases such as obesity, insulin resistance, type 2 diabetes, hyperglycemia, dyslipidemia, and metabolic syndrome due to changes in body weight, increased fasting blood sugar, and cholesterol [4,5]. In addition, mRNA expression of genes, such as peroxisome proliferator-activated receptor-γ (PPARγ), CCAAT/enhancer-binding protein-α (C/EBPα), and leptin, is known increase in obesity [6]. Adipose tissue plays an important role in the immune response, contributing to a chronic low-grade inflammation process linked to tumor development through adipokine secretion molecules, hormone (leptin), growth factors, and proinflammatory cytokines, proliferation, angiogenesis, and expression process [7]. Leptin participates in endocrine metabolism as well as the regulation of appetite and energy expenditure. Recent data have emphasized the brain’s ability to control the complex mechanisms of food intake and storage [8]. The highlight is the value of adipocyte-secreted hormones in obesity and cancer prevention through risk reduction and specific adapted therapies [9]. Recently, in order to prevent and treat obesity, methods such as diet and exercise, as well as next-generation anti-obesity drugs, are being developed [10]. Traditionally, various medicinal plants have been used as treatments for diseases, and various medicinal plants were known to have an effect on obesity [11]. Many studies have scientifically proven that plant extracts can be potential preventive and therapeutic agents for obesity because physiologically active compounds in plant extracts possess anti-obesity effects [12]. Medicinal plants with antioxidant and anti-obesity effects include *Moringa oleifera*, *Solenostemma argel*, and *Salvia* species [13]. The use of natural therapies for weight loss has increased based on their reliability, safety, and cost, compared to synthetic drugs or surgical procedures that have side effects [14]. Among many medicinal plants, annual wormwood (AW: *Artemisia annua* L.) is an annual plant belonging to the *Asteraceae* family that is known for its anti-inflammatory, anti-cancer, antibacterial, and anti-obesity effects and can be found in some parts of Asia, including Korea and China [15]. Compounds, such as ketone, camphor, and 1,8-cineole, exist in AW essential oil [16], and extracts of AW are reported to have excellent antioxidant capacity [17]. In addition, AW has been reported to contribute to the prevention of obesity, including compounds with various anti-obesity activities, such as artemisinic acid, rhamnetin, myricetin, and sabinene [18,19,20]. AW is used as livestock feed and raw material for medicine and cosmetics [21,22].

Glasswort (GW: *Salicornia europaea*) is a halophyte belonging to the pemphigus family *Chenopodiaceae*, which grows on the coasts of temperate and subtropical regions [23]. GW is known to have various functionalities such as antioxidant, anti-cancer, and anti-obesity [24,25]. Chemical constituents such as sterols, quinic acid derivatives, flavonoid derivatives, triterpenoid saponins, and pentadecyl ferulate are present in GW, and some of them have antioxidant activity [26]. In addition, there are various anti-obesity compounds such as trans-ferulic acid (TFA) and isorhamnetin 3-O-β-D-glucopyranoside in GW, which are known to regulate adipogenesis and differentiation inhibition [27,28]. GW is applied as a sodium substitute to various foods such as in baking and sausage making [29,30]. Some of the medicinal herbs used in traditional medicine were biologically activated through the biotransformation of bacteria through fermentation [31]. It is known that fermentation induces the structural destruction of plant cell walls in plant foods and induces the release or synthesis of various antioxidant compounds, especially increasing the amount of phenols and flavonoids due to microbial hydrolysis [32]. As such, AW and GW have been studied for their various physiological activities, but most of them focused on antioxidant and anti-cancer effects. In particular, there are fewer studies on the anti-obesity effects of fermented natural products, and studies comparing anti-obesity effects, physiological activities, and metabolomes before and after fermentation have not been reported.

This study was conducted to elucidate the prospective substances for anti-obesity and to investigate the effects of these medicinal plants as functional additives, as well as natural medicaments. Therefore, the metabolome changes in the medicinal plants AW and GW before and after fermentation, and the differentiation inhibitory ability using 3T3-L1 adipocytes in vitro, were confirmed. In addition, to evaluate the possibility of obesity prevention, male C57BL/6 mice were fed the HFD supplemented with AW and GW.

## 2. Materials and Methods

### 2.1. Preparation of Medicinal Plants

Annual wormwood (AW) and glasswort (GW) were purchased in powder form from a Korean food company and stored in a refrigerator at 4 °C before use (AW: Ingreen Co., Ltd., Gangwha-do, Republic of Korea; GW: Suncheon Bay Hamcho Agricultural Cooperative Corporation, Suncheon-si, Jeollanam-do, Republic of Korea).

### 2.2. Fermentation of Medicinal Plants

The medicinal plant powder, 5% (*w/v*) of AW and GW was added separately to one-fifth diluted Man Rogosa Sharpe (MRS) broth and Bacillus minimal medium (BMM) for the fermentation. Then, pH was adjusted to 7.0 ± 0.5 and sterilized at 121 °C for 15 min. For fermentation, 1% (*v/v*) of *Lactobacillus plantarum* SK3494 and *Enterococcus faecium* SK4369, which was isolated from natural extract of each plant [33,34], was inoculated into the respective medium. The AW and GW were fermented for 16 h at 37 °C using a shaking incubator (BF-60SIRL, Biofree, Seoul, Republic of Korea) under shaking of 100 rpm.

### 2.3. Extraction of Fermented Plants

Before and after fermentation, solution was collected at each time point (0 and 16 h) and stored at −20 °C. After fermentation, ultra-pure water was added to each solution at a rate of 70% and then boiled at 100 °C for 15 min using a water bath (Wise Bath, Seoul, Republic of Korea). Extracted solutions were cooled to room temperature and centrifuged at 14,500 rpm for 15 min (Mega 17R; Hanil, Seoul, Republic of Korea). The collected supernatants were freeze-dried using a freeze dryer (UniFreeze FD-8, Daihan Scientific Ltd., Seoul, Republic of Korea) and stored in a deep freezer at −80 °C for future experiments.

### 2.4. Chemicals and Reagents

The HPLC-grade water and methanol were purchased from Fisher Scientific (Pittsburgh, PA, USA). Formic acid was purchased from Sigma-Aldrich (St. Louis, MO, USA).

### 2.5. Preparation of Metabolic Extracts

The freeze-dried supernatants of non-fermented and fermented samples from AW and GW were extracted using 75% methanol (50 mg/mL). The mixtures were sonicated for 30 min and incubated for 24 h under deep-freezing (−20 °C) conditions. Each of the samples was centrifuged at 11,000 rpm for 10 min, and the collected supernatant was filtered through a 0.22 μm polytetrafluoroethylene filter and dried using a speed vacuum concentrator (Biotron, Seoul, Republic of Korea). The dried samples were reconstituted with 75% methanol to a final concentration of 20 mg/mL, to be used for instrument analysis.

### 2.6. UHPLC–LTQ–Orbitrap–MS Profiling

Metabolite profiling of non-fermented and fermented extracts of AW and GW was preformed using UHPLC–LTQ–Orbitrap–MS/MS. The 2-chloro-phenylalanine (1.5 µg/mL) was used as an internal standard (IS). A sample of 5 μL was injected into a UHPLC system equipped with a Vanquish binary pump H system (Thermo Fisher Scientific, Waltham, MA, USA) coupled with auto-sampler and column compartment at the flow rate of 3 mL/min. Chromatographic separation was performed on a Phenomenex KINETEX^®^ C18 column (100 mm × 2.1 mm, 1.7 μm particle size: Torrance, CA, USA). The mobile phase, 0.1% formic acid in water (A) and 0.1% formic acid in acetonitrile (B), was used in ESI negative mode. The gradient parameters were set as follows: 5% solvent B was maintained initially for 1 min, followed by a linear increase to 100% solvent B over 9 min and then sustained at 100% solvent B for 1 min, with a gradual decrease to 5% solvent B over 3 min. The total run time was 14 min. The column temperature was set to 40 °C, the flow rate was 0.3 mL/min, and the injection volume was 5 μL. The MS data were collected in the range of 100–1000 *m/z* (under negative- and positive-ion modes) using an Orbitrap Velos ProTM system, which was combined with an ion trap mass spectrometer (Thermo Fisher Scientific, Waltham, MA, USA) coupled with an HESI-II probe. The probe heater and capillary temperatures were set to 300 °C and 350 °C, respectively. The capillary voltage was set to 2.5 kV in negative mode (positive mode: 3.7 Kv).

### 2.7. Cell Viability Assay

Cell viability of the plant extracts in 3T3-L1 preadipocytes was measured using cell count kit-8 (WST-8/CCK8, Abcam, ab228554), according to the manufacturer’s instructions. The 3T3-L1 preadipocytes were seeded at 1 × 10^4^ cells in a 96-well plate (SPL, SPL30096) and cultured overnight in a CO_2_ incubator at 37 °C. After incubation, the cells were treated with various concentrations (0.2~50 µg/mL) of plant extracts dissolved in 10 mg/mL DMSO solution and cultured for 72 h. Then, 10 μL of cell count kit-8 (WST-8/CCK8, Abcam, ab228554) reagent was added, and after 30 min, absorbance was measured at 460 nm using a microplate reader. The cell viability was calculated using the following equation:

Cell viability (%) = (OD _Sample_ − OD _Media_)/(OD _DMSO solution_ − OD _Media_) × 100

### 2.8. Oil Red O Staining

Oil Red O [35] staining was performed to confirm intracellular lipid droplet production. Differentiated 3T3-L1 cells were washed with PBS and fixed with 10% formalin for 30 min. After washing, 60% isopropanol and 0.5% ORO solution (Sigma-Aldrich, St. Louis, MO, USA) were added to the fixed cells and stained for 20 min at room temperature. Then, the cell was washed with distilled water, dried at 37 °C, and added to 200 μL of isopropanol to dissolve intracellular ORO solution; then, 100 μL of each was transferred to a 96-well plate, and absorbance was measured at 540 nm using a microplate reader (Synergy 2, BioTek Instruments Inc., Winooski, VT, USA). The ability to inhibit adipocyte differentiation of fermented AW and GW was confirmed for each concentration of 3.1~50 μg/mL.

### 2.9. Reverse Transcription-Polymerase Chain Reaction (RT-PCR)

Reverse transcription-polymerase chain reaction (RT-PCR) was performed to confirm the expression of regulators in fat metabolism using test substances in differentiated 3T3-L1 cells. Changes in mRNA levels of CCAAT/enhancer-binding protein-alpha (C/EBPα), peroxisome proliferator-activated receptor-γ (PPARγ), leptin, and Fas, regulators involved in fat metabolism, were analyzed using RT-PCR and shown in Table 1. Total RNA was isolated using TRI reagent (Sigma, St. Louis, MO, USA), cDNA was synthesized using Power cDNA synthesis kit (iNtRON, Seoul, Republic of Korea), according to the manufacturer’s instructions, and real-time PCR was performed using SYBR green and each primer. PCR was performed using a real-time PCR machine (Corbett, Mortlake, Australia), and gene expression was quantified and analyzed using Rotor-Gene Q Series Software 2.3.1. (Qiagen, Hilden, Germany).

### 2.10. Animals and Diet

The animal experimentation was approved by the NDIC Co., Ltd. Institutional Animal Care and Use Committee (IACUC), Gyeonggi-do, Republic of Korea, in accordance with the guidelines of IACUC (N2021001). Six-week-old male C57BL/6 mice (20~21 g) were obtained from ORIENTBIO Inc., Republic of Korea. The animals were housed in a polycarbonate breeding box under a controlled environment (temperature: 21 ± 2 °C; humidity: 50 ± 20%; lighting time: 12 h/day) and were freely fed food and water. Mice were placed into 6 groups of 8 mice each: normal diet (ND, TEKLAD Certified Global 18% Protein Rodent DIET 2918C, Harlan TEKLAD, Madison, WI, USA); high-fat diet (HFD, D12492; 5.24 kcal/g with 60% of fat-, 20% of protein-, and 20% of carbohydrate-derived calories, Research DIETS Inc., New Brunswick, NJ, USA); HFD supplemented with fermented 100 mg/kg AW extract (AW 100); HFD supplemented with fermented 300 mg/kg AW extract (AW 300); HFD supplemented with fermented 100 mg/kg GW extract (GW 100); and HFD supplemented with fermented 300 mg/kg GW extract (GW 300). Oral administration was performed once a day, and the body weight of the experimental animals was measured once a week for 12 weeks. Feed intake was measured once a week after the test substance was administered. The food efficiency ratio was calculated as FER = total body weight gain (g)/total food intake (g).

### 2.11. Sample Preparation and Treatment

For autopsy, after the inhalational anesthesia with isoflurane, blood was collected through the abdominal vena cava and the liver was removed. The extracted liver was washed with physiological saline, dried with a filter paper, and then the absolute weight was measured using an electronic balance. After calculating the absolute weights, a portion of liver was fixed in 10% neutral buffered formalin for histological examination. The remaining liver was rapidly frozen for hepatic triglyceride (Hepatic TG) analysis and placed in deep freezer (Model 706, Thermo Fisher Scientific Inc., Clevelang, OH, USA) below −70 °C. Blood collected from the abdominal vena cava was placed in a 0.6 mL SST tube (Microtainer, BD, USA), completely solidified, centrifuged at 4 °C at 5000 rpm for 15 min, put into a 1.5 mL tube, and stored in deep freezer (Model 706, Thermo Fisher Scientific Inc., USA) below −70 °C.

### 2.12. Histological Analysis

For histological analysis, liver was fixed in 10% neutral buffered formalin. The fixed tissue was prepared as a paraffin block through a general tissue-processing process and sectioned. The remaining tissues were stored in 10% neutral buffered formalin solution. Hematoxylin and Eosin (H&E) staining was performed on thin slices of liver tissue. The accumulation of lipid droplets in the liver tissue and the degree of adipose inflammation were analyzed using the evaluation method used in the non-alcoholic steatosis model. Non-alcoholic fatty liver disease activity score [13] evaluation, which is a synthesis of five broad categories (steatosis, inflammation, hepatocellular injury, fibrosis, and miscellaneous features), was evaluated on H&E-stained liver tissue slides using the method described in a previous study [36].

### 2.13. Serum and Hepatic Triglyceride Analysis

At the time of autopsy, serum was collected from the abdominal vena cava. Aspartate aminotransferase (AST), alanine aminotransferase (ALT), glucose [24], total cholesterol (T-Chol), triglycerides (TGs), HDL-cholesterol (HDL-C), and LDL-cholesterol (LDL-C) were analyzed using a blood chemistry analyzer (AU480, Beckman Coulter, Germany) using serum stored in deep freezer. Fasting blood insulin levels were measured using the Rat/Mouse Insulin ELISA Kit (EZRMI-13K, Millipore, MA, USA), according to the manufacturer’s instructions, with the separated serum collected from the abdominal vena cava during autopsy and placed in a 0.6 mL SST tube (Microtainer, BD, USA). The remaining serum was stored in deep freezer. At necropsy, 200 mg of liver tissue collected was weighed and washed with normal physiological saline. After adding 1 mL of PBS containing 1% Triton X-100 per 200 mg of liver tissue, homogenization was performed. The liver homogenate was centrifuged at 10,000 rpm at 4 °C for 10 min to separate the supernatant, and then the TG content in the liver tissue was measured with a microplate reader (570 nm) using a TG quantification kit (Cell Biolabs, STA-396, San Diego, CA, USA).

### 2.14. Data Processing and Statistical Analysis

UHPLC–LTQ–Orbitrap–MS raw data files were converted to NetCDF (*.cdf) using Thermo X caliber software (version 2.1, Thermo Fisher Scientific). Retention time correction, peak detection, and alignment were processed using the Metalign software package (http://www.metalign.nl, accessed on 15 November 2021). Metabolites were tentatively identified based on various data comparing mass fragment patterns, retention time, and MS analysis data with standard compounds under identical conditions and in commercial databases, such as the National Institutes of Standards and Technology Library (version 2.0, 2011, FairCom, Gaithersburg, MD, USA), pubchem (https://pubchem.ncbi.nlm.nih.gov/accessed on 1 December 2021), chemspider (http://www.chemspider.com/ on 1 December 2021), and the Human Metabolome Database (HMDB; https://hmdb.ca/ accessed on 1 December 2021). Statistical analysis was performed using SIMCA-P+ software (version 12.0, Umetrics, Umea, Sweden) based on partial least squares discriminant analysis (PLS-DA) modeling to determine metabolite differences between different incubation times. The peaks were selected based on variable importance in the projection (VIP) values and significant differences were determined using analysis of variance (ANOVA). In cell, experiment results were expressed as the mean values ± standard deviations, and significance was tested using the paired-comparison t-test of IBM SPSS Statistics 25 for Windows (IBM, New York, NY, USA) (*p* < 0.05). For multiple comparison, Duncan’s multiple range test was used after comparison test with one-way ANOVA, and significance was verified at *p* < 0.05 level. All results obtained in the mouse experiment were expressed as mean values ± SD, and body weight and weight gain were tested using one-way ANOVA using IBM SPSS Statistics 25 for Windows (IBM, New York, NY, USA), and Fisher’s Least Significant Difference (LSD) test was performed (*p* ≤ 0.05; *p* ≤ 0.01).

## 3. Results

### 3.1. Multivariate Analysis in Annual Wormwood and Glasswort with LAB-Mediated Fermentation

**Figure 1:**
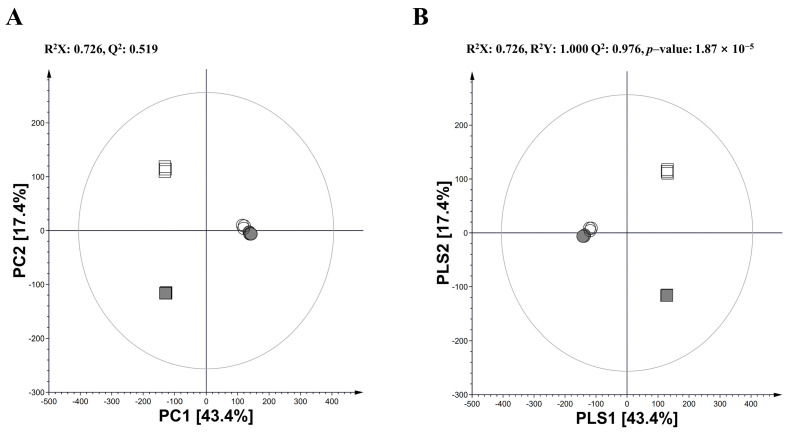
(**A**) PCA score plot and (**B**) PLS-DA score plot derived from UHPLC–LTQ–Orbitrap–MS/MS datasets for non-fermented and fermented extracts of annual wormwood (AW) and glasswort (GW). (■: AN, non-fermentation of AW; □: AF, fermentation of AW with *L. plantarum*; ●: GN, non-fermentation of GW; and ○: GF, fermentation of GW with *Ec. Faecium*).

Non-fermented and fermented extracts of AW and GW were analyzed with UHPLC–MS/MS combined with multivariate analysis on the ingredients to investigate the metabolic change using LAB-mediated fermentation. As a result of principal component analysis (PCA), PC1 (43.4%) and PC2 (17.4%) showed a clear separation of four kinds of extracts, reflecting a metabolic difference between plant species and fermentation (Figure 1A). Similar cluster patterns were also observed in the partial least squares discriminant analysis (PLS-DA) model with a high predictive ability (Q2 = 0.976) as well as a considerable significance metric (*p* = 1.87 × 10^−5^) (Figure 1B).

Based on the PLS-DA model for profiling the dataset, we selected the significantly discriminant metabolites using variable importance in projection (VIP) values > 1.0 and *p* > 0.05. A total of forty-two significantly discriminant metabolites were selected, and thirty-nine of them were tentatively identified including three peptides, two organic acids, six fatty acids, eight phenolic acids and derivatives, seven quinic acids and derivatives, seven benzoic acids and derivatives, and four flavonoids, among two others (Table 2). Plant extracts are rich sources of volatile terpenoids and phenolic compounds with complex mixtures of organic framework-added functional groups including alcohol, aldehydes, esters, ethers, ketones, and phenols. It is used as an analgesic, antibacterial, antidepressant, antimicrobial, and antioxidant agent [22,26]. Polyphenolic compounds are one of the most critical ingredients related to free radical scavenging activity in medicinal plants. They exhibit various biological properties, such as antioxidant, cardioprotective, anti-mutagenic, antibacterial, and anti-inflammatory activities [24].

### 3.2. Relative Metabolite Abundance in Annual Wormwood and Glasswort with LAB-Mediated Fermentation

**Figure 2:**
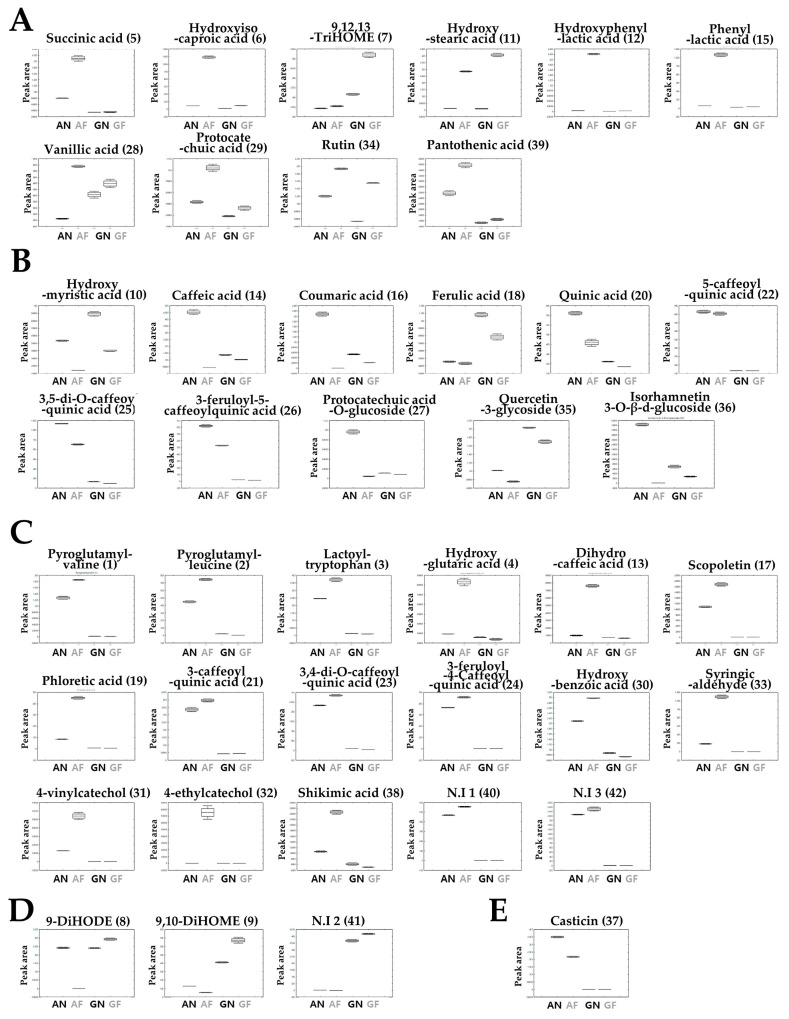
Box-and-whisker plots illustrating the relative abundance of metabolite levels from the fermentation of AW and GW. (**A**) Increased and (**B**) decreased metabolites in both AF and GF. (**C**) Increased and (**E**) decreased metabolites only in the AF group. (**D**) Increased metabolites only in the GF group. (Line: mean; box: standard error; whisker: standard deviation.)

The relative abundance of the significantly discriminant metabolites are shown in the box-and-whisker plots (Figure 2). We observed distinct metabolic change from LAB-mediated fermentation in AW and GW. Several metabolites showed increased or decreased patterns from fermentation in both AF and GF.

The relative contents of several metabolites in both AF and SF groups were higher than each non-fermented group, including succinic acid (5), hydroxyisocaproic acid (6), 9,12,13-TriHOME (7), hydroxystearic acid (11), hydroxyphenyllactic acid (12), phenyllactic acid (15), vanillic acid (27), protocatechuic acid (28), rutin (34), and pantothenic acid (39) (Figure 2A). On the other hand, some metabolites showed lower concentrations from fermentation, that is hydroxymyristic acid (10), caffeic acid (14), coumaric acid (16), ferulic acid (18), quinic acid (20), 5-caffeoylquinic acid (22), 3-5-dicaffeoylquinic acid (25), 3,5-feruloyl-5-caffeoylquinic acid (26), protocatechuic acid-O-glucoside (27), quercetin-3-glycoside (35), and isorhamnetin-3-glucoside (36) (Figure 2B). Some metabolites showed different patterns for each fermented group. Unlike the GF group, the AF group showed a decrease in casticin (37) and an increase in pyroglutamyl-valine (1), pyroglutamyl-leucine (2), lactotyl-tryptophan (3), hydroxyglutaric acid (5), dihydrocaffeic acid (13), scopoletin (17), phloretic acid (19), 3-caffeoylquinic acid (21), 3,4-dicaffeoylquinic acid (23), 3-feruloyl-4-caffeoylquinic acid (24), hydroxybenzoic acid (30), syringic aldehyde (33), 4-ethylcatechol (32), 4-vinylcatechol (31), shikimic acid (38), and two non-identified metabolites (40, 42) (Figure 2C). Relatedly, 9-DiHODE (8), 9,10-DiHOME (9), and N.I 2 (41) increased only in SF (Figure 2D).

### 3.3. Phenolic Acid Degradation Pathways by Biotransformation of Plant Substrates Using LAB

**Figure 3:**
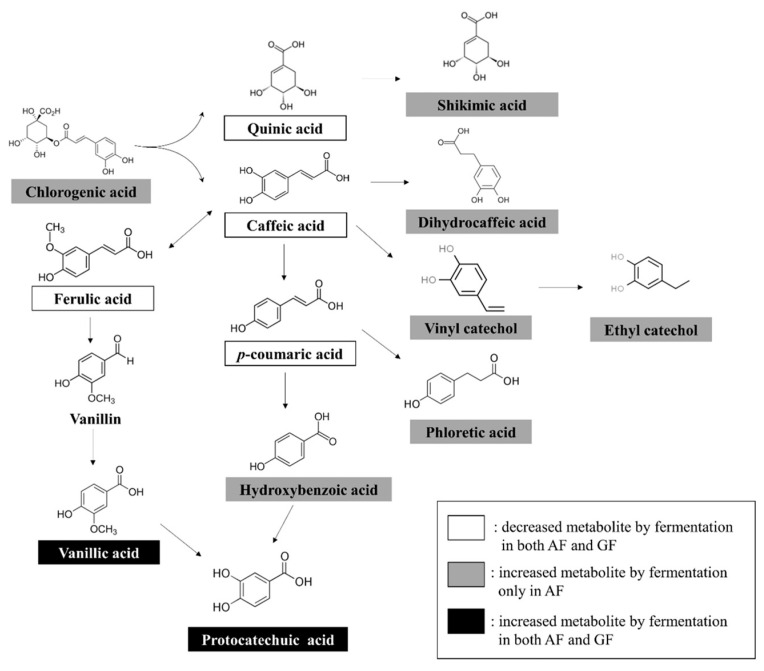
Schematic representation of the degradation pathways of phenolic acids from fermentation of AW and GW.

To investigate the biotransformation of plant substrates, we focused on the phenolic acid degradation pathway in annual wormwood and glasswort. Among the significantly discriminant metabolites, thirteen compounds related to phenolic acid degradation from LAB were selected to construct a metabolic pathway. Chlorogenic-acid-derived caffeic acid, quinic acid, *p*-coumaric acid, and ferulic acid showed a common decrease in both fermentation groups. The contents of metabolites produced by phenolic acid degradation in the AF group were all increased, including shikimic acid, dihydrocaffeic acid, vinyl catechol, ethyl catechol, phloretic acid, hydroxybenzoic acid, protocatechuic acid, and vanillic acid. The SF group also showed an increase in vanillic acid and protocatechuic acid in the phenolic acid degradation pathway (Figure 3).

### 3.4. Inhibition of 3T3-L1 Adipocyte Differentiation

**Figure 4:**
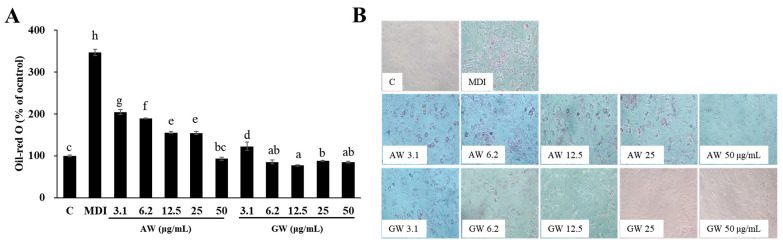
Inhibitory ability of 3T3-L1 adipocyte differentiation from fermented AW and GW extracts. (**A**) Test with oil red O staining method. (**B**) Using an optical microscope. All data are expressed as mean ± SD. Significant differences were determined by Duncan’s test after one-way ANOVA and expressed in letters (a–h) (*p* < 0.05). C: Control; MDI: differentiated control; AW: annual wormwood; GW: glasswort.

To evaluate the anti-obesity effects of these plants, the adipogenesis and fat accumulation were investigated in vitro. Figure 4A shows the adipocyte differentiation inhibitory activity of fermented AW and GW at various concentrations (3.1~50 μg/mL). If the fat accumulation of the control group was 100%, the fermented AW extract showed concentration-dependent adipocyte differentiation inhibitory activity, and at 50 μg/mL, fat accumulation was suppressed as much as the undifferentiated adipocyte control. The GW extract had lower fat accumulation than the control at all concentrations except 3.1 μg/mL and showed great anti-differentiation activity even at low concentrations. Figure 4B shows the results of observing differentiated 3T3-L1 adipocytes treated with fermented AW and GW at various concentrations of 3.1~50 μg/mL under a 100× optical microscope. Both extracts inhibited the differentiation of adipocytes in a concentration-dependent manner, and in the morphology it was observed that the size and number of lipid droplets decreased. Oil Red O staining was performed in vitro, after which the physiological data and effects on adipogenesis were observed based on the histology. In the micrographs, it can be seen that the area stained with Oil Red O was significantly reduced when 3T3-L1 adipocytes were treated with the highest concentration of 50 μg/mL of the AW extract. On the other hand, the GW extract was observed to inhibit the formation of lipid droplets even at low concentrations.

Cells were treated at concentrations ranging from 0.2 to 50 μg/mL to the maximal concentration, showing high survival rates in all treatment groups. AW and GW before and after fermentation at concentrations of 50 μg/mL or less in 3T3-L1 cells did not show cytotoxicity.

### 3.5. Gene Expression Analyzed Using Real-Time PCR (RT-PCR)

**Figure 5:**
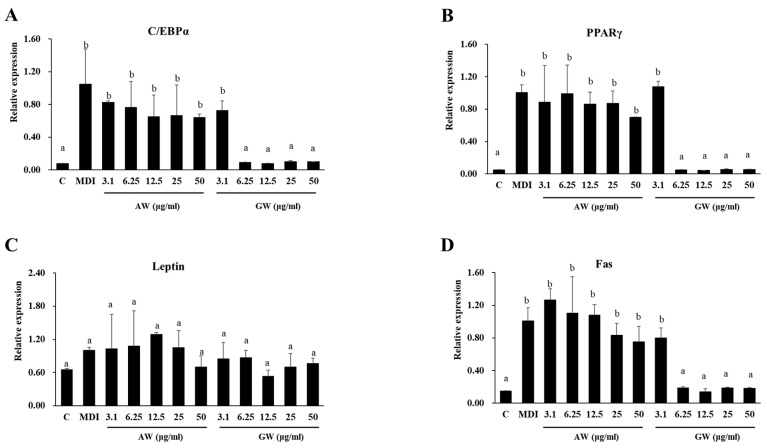
Effect of fermented AW and GW extracts on the mRNA expressions of (**A**) C/EBPα, (**B**) PPARγ, (**C**) leptin, and (**D**) Fas, regulatory factors involved in 3T3-L1 preadipocytes. All data are expressed as mean ± SD. Significant differences were determined by Duncan’s test after one-way ANOVA and expressed in letters (*p* < 0.05). C: Control; MDI: differentiated control; AW: annual wormwood; GW: glasswort.

To test the anti-obesity effect of plant extracts on lipogenesis in adipose tissue, the real-time PCR was analyzed. The effect of the AW and GW extracts on the expression of adipocyte differentiation-related factors (C/EBPα, PPARγ, leptin, and Fas) in 3T3-L1 cells was experimented (Figure 5). PPARγ is a nuclear hormone receptor that plays a major role in regulating the expression of proteins necessary for the development of functional mature adipocytes. C/EBPα plays a synergistic role in terminal adipocyte differentiation. C/EBPα and PPARγ are integrated in the control of lipid and carbohydrate metabolism. In the real-time PCR results, the gene expression of C/EBPα and PPARγ for adipogenesis decreased compared to the MDI group. After adipocyte differentiation (MDI), the expression of all genes, except leptin, increased significantly compared to before differentiation (control). There was no significant difference in gene expression of adipocytes treated with AW extract compared to MDI. On the other hand, when the GW extract was treated at a concentration of 6.2 μg/mL or more, the expression of differentiation-related genes, except for leptin, was significantly reduced compared to MDI. In addition, C/EBPα and PPARγ, which are closely related to each other, showed a similar decrease pattern, and Fas involved in adipogenesis was reduced. In the case of leptin, GW tended to be less produced than AW, but there was no significant difference. As a result, since the GW extract of 6.2 μg/mL or more reduces the expression of adipocyte genes to the same level as the control, it can be assumed to have an inhibitory activity on the differentiation of 3T3-L1 preadipocytes. The effects of the GW extract on the inhibition of adipogenesis were observed more apparently.

### 3.6. Changes in Body and Organ Weight in C57BL/6 Mice

**Figure 6:**
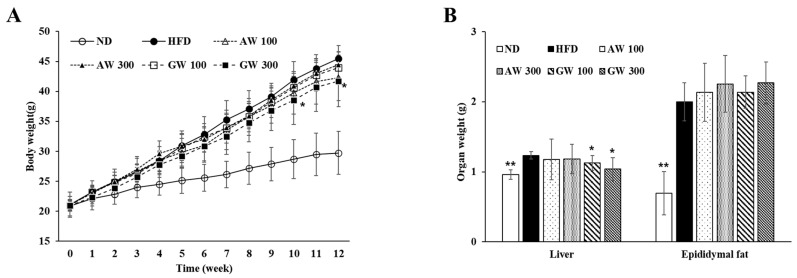
Changes in (**A**) body weight and (**B**) organ weight of C57BL/6 mice during the experiment period. Data are presented as mean ± SD (n = 8). Significant difference from HFD using one-way ANOVA followed by the LSD test: * *p* < 0.05, ** *p* < 0.01. ND: Normal diet; HFD: 60% high-fat diet; AW 100: HFD + fermented annual wormwood (AW) 100 mg/kg; AW 300: HFD + fermented AW 300 mg/kg; GW 100: HFD + fermented glasswort (GW) 100 mg/kg; GW 300: HFD + fermented GW 300 mg/kg.

Figure 6A shows the body weight and organ weight changes for 12 weeks of C57BL/6 mice fed a high-fat diet containing fermented AW and GW extracts at 100 and 300 mg/kg. The body weight of the GW 300 significantly decreased at 10 and 12 weeks compared to the HFD (*p* < 0.05). The final body weight of the ND was 29.73 ± 3.59 g, an increase of 8.86 ± 2.25 g from the initial body weight, whereas the final body weight of the HFD was 45.50 ± 2.15 g, an increase of 24.43 ± 2.26 g from the initial weight. The HFD significantly increased body weight compared to the ND from the second week of the experiment, and a significant weight increase was observed continuously until the end of the test (*p* < 0.01), indicating that obesity was induced in mice due to the high-fat diet. The final body weight of AW 100 among the AW extract treatment groups was 42.26 ± 3.80 g, and there was no significant difference compared to the HFD, but the weight gain was significantly reduced to 21.13 ± 2.26 g (*p* < 0.05). Relatedly, among the GW extract treatment groups, GW 300 had a final body weight of 41.71 ± 4.27 g and a body weight gain of 20.71 ± 3.68 g, which was significantly reduced compared to the HFD (*p* < 0.05). In addition, the body weight of the GW 300 significantly decreased at 10 and 12 weeks compared to the HFD (*p* < 0.05), and the body weight gain significantly decreased at 7, 10, 11, and 12 weeks (*p* < 0.05). The weights of the initial mice were similarly set in the range of 20~21 g. The FER was lowest in the ND and highest in the HFD. Compared to the HFD, the FER of the AW 300 and GW 300 groups was significantly lower (*p* < 0.05). In particular, AW 300 had a small FER even though its weight gain was the second highest after the HFD, suggesting that weight gain was small even though it consumed a lot of food. Therefore, it was considered that the AW and GW extracts affected the weight loss and FERs of C57BL/6 mice. Figure 6B shows the liver weights of C57BL/6 mice fed a high-fat diet containing fermented AW and GW extracts of 100 and 300 mg/kg. Liver weights in the ND were significantly decreased compared to the HFD (*p* < 0.01). The AW 100 and AW 300 had no significant differences. Compared to the HFD, the liver weights of the GW 100 and GW 300 decreased significantly (*p* < 0.05). In conclusion, GW inhibited the accumulation of triglycerides in the liver.

### 3.7. Histological Analysis

**Figure 7:**
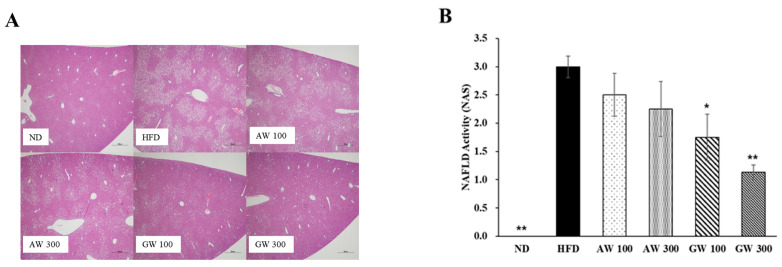
Effect of fermented AW and GW extracts on (**A**) histological features of the liver and (**B**) non-alcoholic fatty liver disease activity score in liver of C57BL/6 mice. Original magnifications: ×40; scale bar: 500 μm. Data are presented as mean ± SD (n = 8). Significant difference from HFD using *t*-test: * *p* < 0.05, ** *p* < 0.01. ND: Normal diet; HFD: 60% high-fat diet; AW 100: HFD + fermented annual wormwood (AW) 100 mg/kg; AW 300: HFD + fermented AW 300 mg/kg; GW 100: HFD + fermented glasswort (GW) 100 mg/kg; GW 300: HFD + fermented GW 300 mg/kg.

In order to analyze non-alcoholic steatosis, using image J program photographed the lipid droplet and inflammatory symptoms in liver tissue. Figure 7A showed the histological feature of the liver based on hematoxylin and cosin staining in rats fed control and HFD. Figure 7A displayed liver tissue images of C57BL/6 mice fed a high-fat diet containing fermented AW and GW extracts (100 and 300 mg/kg). Compared to the ND, it was confirmed that the number of lipid droplets produced in the liver tissue of obese HFD increased. Lipid droplets were less in the liver tissue of C57BL/6 mice fed with the GW extract than with the AW extract. Therefore, it was considered that GW inhibits lipid accumulation in the liver more than AW. The most common cause of chronic disease by excessive fat accumulation in the liver is non-alcoholic fatty liver disease (NAFLD). NAFLD caused that high carbohydrate diet induced serum free fatty acid, thereafter increased the absorption of FFA biosynthesis of TG in liver. NAFLD includes a wide spectrum of liver damage ranging from simple steatosis to non-alcoholic steatohepatitis (NASH), advanced fibrosis, and hepatocellular carcinoma [37]. Figure 7B shows the NAFLD activity score measuring the histological characteristics of non-alcoholic fatty liver disease (NAFLD) in C57BL/6 mice fed a high-fat diet containing fermented AW and GW extracts (100 and 300 mg/kg). The NAS of the C57BL/6 mice fed normal diet (ND) in which obesity was not induced was 0, and NAFLD did not appear, and it was significantly lower than that of the HFD (3.00 ± 0.19) (*p* < 0.01). AW 100 (2.5 ± 0.38) and AW 300 (2.25 ± 0.49) decreased concentration dependently compared to the HFD, but no significant difference occurred. GW 100 (1.75 ± 0.41) and GW 300 (1.13 ± 0.13) also decreased in a concentration-dependent manner with significant differences compared to the HFD (*p* < 0.05, *p* < 0.01).

### 3.8. Serum and Hepatic Triglycerie Analysis

Serum analysis was performed in C57BL/6 mice fed a high-fat diet containing fermented AW and GW extracts at 100 and 300 mg/kg (Table 3). Serum AST and ALT levels are indicators of the liver dysfunction condition, caused by the HFD. AST (163.38 ± 20.60) and ALT (122.50 ± 31.01) values of AW 100 were significantly increased, compared to the HFD (*p* < 0.01, *p* < 0.05). The level of AST in the normal group was 94 U/L, and the HFD G2 group significantly increased; in contrast, in the supplemented AW 300 groups was significantly decreased. The ALT values of the supplemented GW groups were significantly decreased; in particular, the G6HFD 300 (46.63 ± 3.30) groups showed the lowest value of all the other HFD groups (p < 0.05). In the case of blood Glu, those of the ND (118.00 ± 10.49), GW 100 (212.63 ± 12.40), and GW 300 (210.50 ± 17.02) were decreased when compared to the HFD at 226.00 ± 4.76 mg/dL (*p* < 0.01). Blood T-Chol significantly decreased in all groups compared to the HFD at 210.75 ± 8.39 mg/dL. Blood TGs significantly decreased at the GW 300 at 80.00 ± 3.33 mg/dL, compared to the HFD at 97.75 ± 5.31 mg/dL (*p* < 0.01). HDL-C was significantly reduced, compared to the HFD at 97.13 ± 2.73 mg/dL, in the AW 300 and GW 300 at 89.25 ± 2.94 and 91.50 ± 2.17 mg/dL, respectively, treated with high-concentration extract (*p* < 0.05). The GW extract treatment groups, GW 100 14.75 ± 0.70 and GW 300 13.63 ± 0.91 mg/dL, showed reduced LDL-C, compared to the HFD at 18.88 ± 1.33 mg/dL (*p* < 0.05). As a result of the blood test, when obesity was induced, the values of all blood analysis indicators increased. On the other hand, the AW extract reduced T-Chol and HDL-C in C57BL/6 mice, and the fermented GW extract reduced blood ALT, Glu, T-Chol, TGs, HDL-C, and LDL-C. As a result, since AW and GW reduce the levels of ALT, Glu, T-Chol, TGs, HDL-C, and LDL-C in the blood, it is thought that they have the possibility of preventing obesity. Insulin levels in the HFD, in which obesity was induced by eating a high-fat diet, were increased, compared to the ND. Among the plant extract treatment groups, only GW 300 at 2.70 ± 0.66 ng/mL significantly reduced blood insulin levels (*p* < 0.01), compared to the HFD at 5.55 ± 0.96 ng/mL. When obesity was induced, hepatic TGs increased, and the AW 300 at 103.31 ± 89.76 µg/mg tissue decreased, compared to the HFD at 145.66 ± 56.7 µg/mg tissue, but there was no significant difference. Hepatic TG levels tended to decrease in a dose-dependent manner in the GW extract treatment group, and GW 300 decreased significantly, compared to the HFD (*p* < 0.05). Therefore, it can be assumed that GW 300 suppressed the accumulation of triglycerides in the liver.

## 4. Discussion

Obesity is a major public health problem with high prevalence worldwide and is accompanied by various diseases such as diabetes, hyperglycemia, dyslipidemia, and metabolic syndrome [5]. Treatments are being developed to prevent and treat obesity, but side effects such as nausea and diarrhea exist [9]. In order to develop plant-derived functional materials with fewer side effects and superior anti-obesity effects than drugs, this study aimed to reveal the anti-obesity activity of fermented annual wormwood (AW) and glasswort (GW) at both the in vitro and in vivo levels. In addition, metabolome changes were verified for the difference before and after fermentation. AW (*Artemisia annua* L.) is a common type of wormwood native to temperate Asia but naturalized worldwide and belonging to the family *Asteraceae* [15]. Its bioactive derivatives control ROS, DNA damage, DNA repair, cell cycle arrest, apoptosis, inflammatory response, angiogenesis, and multiple signaling pathways, showing an anti-cancer effect [38]. In addition, it has an anti-hyperglycemic effect and reduces insulin resistance by increasing adiponectin secretion in adipocytes [35].

GW (*Salicornia herbacea* L.) belongs to *Chenopodiaceae* and has been used as a food ingredient or as a folk remedy for improving intestinal function, indigestion, gastrointestinal disease, kidney disease, and diabetes [39]. GW is seaweed that contains physiologically active substances, such as dietary fiber, and minerals such as potassium, magnesium, and calcium that stimulate intestinal movement. GW is known to have beneficial effects on obesity by reducing body weight and fat levels and improving blood lipids [40].

The bacteria used for AW fermentation are *Lactobacillus plantarum*, a lactic acid bacterium [41]. It is known that pH decreases by producing lactic acid as a fermentation metabolite [42]. At this time, as the fermentation progresses, the pH decreases and the acidity increases, which is considered to reduce the activity of *Lactobacillus plantarum*. GW showed a small change in pH during fermentation, an increase in viable cell count, and then a tendency to die after a certain period of time, which was similar to previous studies [33]. Since *Enterococcus faecium*, the strain used for GW fermentation, mainly uses carbohydrates as an energy source, it is thought that the death occurred due to a lack of carbohydrates as fermentation proceeded [43].

AW and GW contain phenolic compounds, and there is no difference in physiological activity before and after fermentation. It is true that fermentation is known as one of the ways to improve the physiological activity of natural products, but it is difficult to see that some phenols are unconditionally improved due to small changes [44].

Untargeted metabolomics were employed to investigate metabolic change using LAB-mediated fermentation in AW and GW. Among the discriminant metabolites, we observed the relative abundance of several metabolites related to bacterial biotransformation of plant-derived compounds such as flavonoids and phenolic acids. Flavonoids in plants are major secondary metabolites, mainly in the form of flavonoid glycosides [45]. Flavonoid glycosides can be decomposed into aglycone and phenolic acid by microorganisms. Some LAB have enzymes that convert flavonoid glycosides such as α-l-rhamnosidase and β-d-glucosidase, but these abilities vary among specific species and strains. In this study, the reduction of quecetin-3-glycoside and isorhamnetin-3-glycoside using fermentation was observed, but the types of aglycone and flavonol produced from bioconversion could not be identified. On the other hand, the content of rutin tended to increase through fermentation. It has been reported that there is a difference in the conversion ability of rutin for each strain. In the study of buckwheat fermented by 14 LAB strains, most of them decreased the content of the rutin, but some strains increased [46]. Rutin is known to have various pharmacological activities such as antimicrobial, anti-inflammatory, and anti-cancer effects [47,48]. In addition, rutin administration in high-fat diet mice was affected, affecting the intestinal bacteria and anti-obesity effect [49].

Bacterial degradation of phenolic compounds has also been reported along with metabolic changes. The reaction of phenolic acid decarboxylase (PAD) and reductase in LAB decreased phenolic acid as a substrate and increased some benzoic acid as a product [50,51,52]. We also observed the fermentation patterns of metabolites in the degradation pathways of quinic acid, caffeic acid, *p*-coumaric acid, and ferulic acid. As a clear reduction of phenolic acid, we identified the products of eight benzoic acid related to four kinds of phenolic acid in the AF group and only two compounds (vanillic acid and protocatechuic acid) related to ferulic acid in the GF group. The converted metabolites derived from phenolic acid showed high bioavailability in human and health benefits [53,54]. Dihydrocaffeic acid and the catechol group converted from caffeic acid were reported as potent antioxidant properties caused by the scavenging of intracellular reactive oxygen species [41,55,56]. Relatedly, phloretic acid converted from p-coumaric acid was one of the substances that recently attracted attention as physiological activities, such as protecting the host from influenza infection by regulating the immune balance, that have been reported [57,58]. Hydroxybenzoic acid and its derivatives are known to exhibit various physiological activities such as anti-obesity, antibacterial, anti-mutagenic, anti-algae, anti-inflammatory, and antioxidant activities [59,60,61]. In addition, protocatechuic acid was also reported to have various effects such as anti-obesity, anti-inflammatory, and antioxidant effects through in vivo and in vitro tests [62,63]. Vanillic acid is a substance used pharmacologically with various physiological activities such as anti-obesity, antioxidant, anti-cancer, antifungal, and liver protection [64,65]. 

Additionally, we observed the production of several metabolites related to amino acid and fatty acid metabolism of LAB. These metabolites were presumed to be produced using nutrients in the medium rather than compounds derived from plants. Phenyllactic acid, hydroxyphenyllactic acid, and hydroxyisocaproic acid were produced by amino acid catabolism, which was phenylalanine, tyrosine, and leucine, respectively [66,67]. In fatty acid metabolism, oleic acid and linoleic acid were metabolized to hydroxystearic acid, azelaic acid, and hydroxyoctadecanoic acid [68,69]. Among these increased metabolites, hydroxy fatty acids and phenylpropanoids produced by LAB have been reported to have antifungal activity that can be useful to biopreservation [70,71,72]. On the other hand, some dipeptides (pyroglutamyl-valine, pyroglutamyl-leucine, and lactoyl-tryptophan) increased only in the AF group. Those compounds included the taste active peptide derivatives in food fermentation of LAB [73]. An anti-inflammatory effect and attenuating dysbiosis in vivo has been reported for pyroglutamyl-leucine [74,75]. Similarly, some oxylipins (9-DiHODE, 9,10-DiHOME, and 9,12,13-TriHOME) dramatically increased in the GF group, which are well-known as bioactive lipids for their anti-inflammatory and antioxidant effects [76,77].

Relatedly, the anti-obesity effect of fermented AW and GW was confirmed in vitro using 3T3-L1 cells, which can differentiate between fibroblasts and adipocytes and are widely used in adipogenesis and biochemical studies of adipocytes [78]. There was no cytotoxicity because both AW and GW showed a high survival rate up to 50 μg/mL. Lipid droplet generation can be confirmed by staining triglyceride and cholesterol ester accumulated due to lipolysis using Oil Red O staining reagent, and the degree of fat differentiation can be quantified by dissolving it and measuring absorbance [79,80]. Both the fermented AW and GW extracts inhibited the differentiation of adipocytes in a concentration-dependent manner, and it was morphologically observed that the size and number of lipid droplets produced when adipocytes differentiated and accumulated fat decreased. Excessive lipid accumulation and formation of lipid droplets cause obesity [81], and it was confirmed that GW suppressed the formation of lipid droplets even at low concentrations when compared to AW. Therefore, it can be said that fermented GW has the potential for preventing obesity.

During adipogenesis, several types of genes and transcription factors were regulated. In this experiment, the effects of fermented AW and GW extracts on the expression of adipocyte differentiation-related factors C/EBPα, PPARγ, leptin, and Fas in 3T3-L1 cells were investigated. C/EBPα and PPARγ are specific transcription factors expressed in adipocytes, and the activities of the two regulators were enhanced in the early stages of adipogenesis and promote adipogenesis [82]. When adipocytes were compared before and after differentiation, C/EBPα and PPARγ significantly increased after differentiation, and transcription factors were suppressed in the GW group, similarly to before differentiation. Leptin, a protein produced and secreted by adipocytes, increased as fat accumulation increased and is a hormone that induces appetite suppression to control obesity. Leptin levels increased after differentiation, but there was no significant difference. The level of leptin in AW was similar to that after differentiation, but in the case of GW, it slightly decreased. Since leptin secretion increased with adipocyte size and fat accumulation, it was thought that the level of leptin was low in GW where fat accumulation was suppressed [83]. When the expression of Fas, which is involved in lipid synthesis, transport, and storage, was inhibited, adipogenesis and triglyceride accumulation were reduced [84]. The expression of Fas increased after differentiation, and GW suppressed the expression to the same level as the control group. Demineralized glasswort and annual wormwood leaves, which have been shown to have an inhibitory effect on the differentiation of adipocytes, and fermented lemons showed similar gene expression inhibitory activity [28,85,86]. Therefore, 3T3-L1 cells increase the expression of all genes as fat accumulates after differentiation, but GW extract reduces the expression of adipocyte-expressed genes C/EBPα, PPARγ, and Fas, suggesting that the differentiation of adipocytes is inhibited.

The anti-obesity activity was investigated through weight change, histological change, and serum analysis. C57BL/6 mice were used as experimental animals, and they are often used as obesity-prevention models because they lack the leptin gene and cannot control appetite, causing obesity [87]. In this experiment, it was also confirmed that the weight of the high-fat diet (HFD) group continued to increase, and obesity was caused by the high-fat diet. After 12 weeks, the final weight and weight gain (g) of GW 300 decreased compared to the HFD, and the weight gain (g) of AW 100 decreased (*p* < 0.05). The food efficiency ratios (FERs) of the AW 300 group and GW 300 group significantly decreased compared to the HFD (*p* < 0.05). If the FER was small, it can be predicted that obesity was prevented in the high-concentration treatment group of natural products [88].

Obesity refers to an increase in body fat caused by the accumulation of triglycerides in adipose tissue, and it has been reported that the risk of metabolic diseases increases as the abdominal fat content increases [89]. In this experiment, the weight of the liver was measured to confirm the formation of abdominal fat. Similar to the previous study [86], in which C57BL/6 mice fed the HFD gained weight as cholesterol and triglycerides accumulated in the liver, it was confirmed that the liver weight increased in this experiment. The liver weight of mice fed the GW extract significantly decreased compared to the HFD (*p* < 0.05). The GW extract inhibited liver fat accumulation. In addition, when lipids accumulated in the liver as obesity progressed, lipid droplets also accumulated [90]. An obesity-induced HFD increased the number of lipid droplets in liver tissue compared to the ND, and it was confirmed that the number of lipid droplets in GW was smaller than that in AW. Non-alcoholic fatty liver diseases (NAFLDs) are known to be liver damage diseases caused mainly by insulin resistance, accompanied by obesity and dyslipidemia due to the accumulation of triglycerides in hepatocytes [91]. The non-alcoholic fatty liver disease activity score [13] is a method of expressing NAFLD as a score for histological characteristics. Non-alcoholic steatohepatitis (NASH) can be diagnosed if the NAS is five or higher, and “not NASH” if the NAS is less than three [36]. The HFD group has a high risk of NASH with a score of three, and all groups except the HFD group can be diagnosed as not NASH, so it can be said that there is no liver damage. In addition, the content of useful substances having various activities increased by this decomposition. Serum aspartate aminotransferase (AST) and alanine aminotransferase (ALT), biomarkers that can estimate liver health, increased in obesity. Similarly, AST and ALT of an obesity-induced HFD increased significantly compared to the ND. The ALT of the GW 300 group significantly decreased compared to the HFD (*p* < 0.01). On the other hand, AW 100 increased both the AST (*p* < 0.01) and ALT (*p* < 0.05), compared to the HFD, which was contrary to previous studies in which the AST and ALT of the AW extract were lower than those fed a high-fat diet [15,25]. When AST and ALT increased, it can be said that liver damage had occurred. In the present study, two of the 100 AW groups had higher AST and ALT than other animals, suggesting that liver damage may have occurred. Blood glucose [24] was the highest in an obesity-induced HFD and significantly decreased in the GW-treated group, compared to the HFD group (*p* < 0.01). The GLU-lowering effect of GW was similar to a previous study that revealed a hyperglycemia-preventive effect [92]. The total cholesterol (T-Chol) and high-density lipoprotein-cholesterol (HDL-C) of mice fed fermented AW extract significantly decreased when compared to the HFD (*p* < 0.05). The fermented GW extract significantly reduced GUL, T-Chol, triglycerides (TGs) (*p* < 0.01), HDL-C, and LDL-C (*p* < 0.05). GW lowered the lipid concentration in the blood than AW. This result was similar to a previous experiment in which T-Chol, low-density lipoprotein-cholesterol (LDL-C), and TG levels were reduced in mice fed high-fat diets and seaweed [93]. The occurrence of cardiovascular disease can be confirmed by the TC/HDL-C ratio, and it is known that the occurrence of the disease is low when the ratio is low. The TC/HDL-C ratios were ND (1.38), HFD (2.17), AW 100 (2.03), AW 300 (1.99), GW 100 (1.90), and GW 300 (1.78), and ND and GW 300 were low. Therefore, the fermented GW extract can reduce the risk of obesity and cardiovascular disease by reducing blood ALT, GUL, T-Chol, TGs, LDL-C, blood fat, and cholesterol.

Obesity increases insulin resistance, which does not normalize blood glucose levels, and increases the risk of developing type 2 diabetes [94]. In the case of the HFD, insulin resistance was high, but blood sugar increased, so it can be judged that insulin resistance occurred. Among the experimental groups, GW 300 showed a decrease in insulin level (*p* < 0.01), and even though they ate a high-fat diet, the rate of insulin secretion was regulated, and it is thought that the GW extract lowered insulin resistance. When obesity was induced, hepatic triglycerides (hepatic TGs) increased, and among them, the GW 300 group significantly decreased compared to the HFD (*p* < 0.05), and it was found that obesity was suppressed. Glasswort supplementation showed similar results to a previous study [95] that significantly reduced hepatic triglycerides compared to mice fed a high-fat diet.

## 5. Conclusions

In this study, in vitro experiments using 3T3-L1 adipocyte cells and in vivo experiments using C57BL/6 mice were conducted to investigate the anti-obesity effects of fermented annual wormwood (AW) and glasswort (GW). In addition, an analysis of compound changes was investigated comparing before and after fermentation. In particular, phenolic compounds were abundant in AW, and antioxidant activity was also high. In both AW and GW plants, significant metabolite changes from fermentation were observed. The metabolites increased from fermentation were metabolites produced by decomposing the plant substrate and substances produced by strain biosynthesis. Specifically, several substances with reported physiological activity were identified from the degradation of phenolic acid derived from plants using lactic acid bacteria. It is suggested that the compounds involved in the anti-obesity activity of GW can be activated by the biotransformation of fermentation, but detailed research is needed for the exact mechanism.

The AW and GW extracts were not toxic to 3T3-L1 adipocytes up to the maximum concentration of 50 µg/mL, and the plants after fermentation had better ability to inhibit adipocyte differentiation. The fermented AW and GW extracts inhibited the differentiation of adipocytes in a concentration-dependent manner and also reduced the size and number of lipid droplets. In particular, fermented GW inhibited lipid droplet accumulation even at low concentrations and significantly reduced the expression of C/EBPα, PPARγ, and Fas, transcription factors and genes involved in adipogenesis that were expressed during adipocyte differentiation. When C57BL/6 mice were supplemented with fermented AW extract, the body weight, food efficiency ratio (FER), and total blood cholesterol (T-Chol) reduced compared to obese mice. The supplementation of GW extract to C57BL/6 mice prevented liver lipid accumulation and fatty liver as well as weight loss. GW extract also prevented liver disease, reduced blood lipids, and regulated insulin.

Through this study, the anti-obesity activity of glasswort was revealed in vitro and in vivo experiments such as weight loss and lipid accumulation inhibition. Compounds with anti-obesity activity were activated through biotransformation using fermentation. As seen in the results, it is suggested that glasswort and annual wormwood be used for companion animals as well as human health as supplementary food additives for the control of obesity and metabolic disorders. The metabolic extracts from the fermented plants have shown promising anti-obesity effects which need to be substantiated further with large-scale animal studies and cross-sectional population trials with physiological studies.

## Figures and Tables

**Table 1:** Primer sequences of target genes used in the PCR.

Gene	Name	Primer Sequence	Tm (°C)
C/EBPα	mC/EBPα_F	CAA GAA GTC GGT GGA CAA G	55.2
	mC/EBPα_R	GCT TTA TCT CGG CTC TTG C	55.2
PPARγ	mPPARγ_F	GAC ATC CAA GAC AAC CTG CT	55.4
	mPPARγ_R	TGT CAT CTT CTG GAG CAC CT	55.4
Leptin	mLeptin_F	TGA CAC CAA AAC CCT CAT CA	53.4
	mLeptin_R	AGC CCA GGA ATG AAG TCC A	55.2
Fas	mFas_F	AGA GAT CCC GAG ACG CTT CT	57.4
	mFas_R	GCT TGG TCC TTT GAA GTC GAA GA	58.2

**Table 2:** Tentatively identified metabolites in non-fermented and fermented extracts of AW and GW based on the UHPLC–LTQ–Orbitrap–MS/MS analyses.

No.	Tentative Metabolite	VIP ^a^ 1	VIP 2	RT (min) ^b^	MW	Measured Mass	MS/MS Fragments	Molecular Formula	Delta ppm
						**Negative Mode** **^c^** **(*m*/*z*)**			
*Peptides*									
1	Pyroglutamyl-valine	1.47	1.12	2.18	228	227.1046	227 > 183 > 155, 127, 82	C_10_H_15_O_4_N_2_	3.698
2	Pyroglutamyl-leucine	1.43	1.15	4.17	242	241.1202	241 > 197 > 169, 141	C_11_H_17_O_4_N_2_	3.441
3	Lactoyl-tryptophan	1.45	1.13	4.53	276	275.1049	275 > 231, 127 > 109	C_14_H_15_O_4_N_2_	4.325
*Organic acid*									
4	Hydroxyglutaric acid	0.97	1.46	1.06	148	147.0307	147 > 129 > 101, 85	C_5_H_7_O_5_	5.124
5	Succinic Acid	1.17	1.35	1.06	118	117.0200	117 > 99, 73	C_4_H_5_O_4_	6.307
*Fatty acid*									
6	Hydroxyisocaproic acid	0.91	1.50	4.02	132	131.0722	131 > 113, 85	C_6_H_11_O_3_	6.275
7	9,12,13-TriHOME	1.09	0.79	6.59	330	329.2343	329 > 229 > 211, 125	C_18_H_33_O_5_	2.742
8	9-DiHODE	0.96	1.42	7.29	312	311.2241	311 > 293 > 275, 185	C_18_H_31_O_4_	4.297
9	9,10-DHOME	1.36	0.97	7.99	314	313.2393	313 > 295 > 277, 195	C_18_H_33_O_4_	2.737
10	Hydroxymyristic acid	0.92	1.13	8.99	244	243.1974	243 > 225 > 207, 181	C_14_H_27_O_3_	3.257
11	Hydroxystearic acid	0.18	1.03	9.64	300	299.2598	299 > 281, 253 > 249, 225	C_18_H_35_O_3_	2.211
*Phenolic acids and deriatives*									
12	Hydroxyphenyllactic acid	0.89	1.51	2.37	182	181.0522	181 > 163 > 119	C_9_H_9_O_4_	4.462
13	Dihydrocaffeic acid	0.90	1.50	3.54	182	181.0515	181 > 137 > 119, 109	C_9_H_9_O_4_	4.849
14	Caffeic acid	0.60	1.61	4.02	180	179.0360	179 > 135 > 107, 91	C_9_H_7_O_4_	5.574
15	Phenyllactic acid	0.91	1.50	4.56	166	165.0571	165 > 147 > 121,97	C_9_H_9_O_3_	5.226
16	Coumaric acid	0.60	1.60	4.69	164	163.0409	163 > 119 > 91	C_9_H_7_O_3_	5.168
17	Scopoletin	1.42	1.16	4.89	192	191.0358	191 > 177 > 104	C_10_H_7_O_4_	4.177
18	Ferulic acid	1.42	1.01	4.96	194	193.0516	193 > 178, 149 > 134	C_10_H_9_O_4_	5.117
19	Phloretic acid	1.08	1.41	4.97	166	165.0564	165 > 147, 121 > 106, 93	C_9_H_9_O_3_	4.68
*Quinic acid and deriatives*									
20	Quinic acid	1.29	1.26	0.85	192	191.0569	191 > 173, 127, 111, 85	C_7_ H_11_O_6_	4.128
21	3-caffeoylquinic acid	1.50	1.08	2.29	354	353.0894	353 > 191 > 173, 127, 85	C_16_H_17_O_9_	2.222
22	5-caffeoylquinic acid	1.51	1.07	3.85	354	353.0894	353 > 191 > 173, 127, 85	C_16_H_17_O_9_	4.573
23	3,4-di-*O*-caffeoylquinic acid	1.50	1.08	4.39	516	515.1215	515 > 353 > 191, 179	C_25_H_23_O_12_	3.923
24	3-feruloyl-4-caffeoylquinic acid	1.50	1.09	4.78	530	529.1362	529 > 367 > 193	C_26_H_25_O_12_	4.688
25	3,5-di-*O*-caffeoylquinic acid	1.18	0.83	5.03	516	515.1213	515 > 353 > 191, 179, 135	C_25_H_23_O_12_	2.389
26	3-feruloyl-5-caffeoylquinic acid	1.44	1.15	5.48	530	529.1367	529 > 367 > 191, 173	C_26_H_25_O_12_	2.855
*Benzoic acid and derivatives*									
27	Protocatechuic acid-*O*-glucoside	0.77	1.56	1.51	316	315.0735	315 > 153 > 123, 109	C_13_H_15_O_9_	4.332
28	Vanillic acid	0.11	1.66	1.61	168	167.0359	167 > 152, 149 > 121	C_8_H_7_O_4_	5.675
29	Protocatechuic acid	1.13	1.37	1.96	154	153.0201	153 > 138, 109 > 81	C_7_H_5_O_4_	5.281
30	Hydroxybenzoic acid	1.42	1.15	2.83	138	137.0251	137 > 93	C_7_H_5_O_3_	5.201
31	4-vinylcatechol	1.14	1.36	5.29	136	135.0452	135 > 107, 91	C_8_H_7_O_2_	5.163
32	4-ethylcatechol	0.88	1.50	5.56	138	137.0615	137 > 93	C_8_H_9_O_2_	4.721
33	Syringic aldehyde	1.05	1.43	6.09	182	181.0513	181 > 166 > 138	C_9_H_9_O_4_	3.689
*Flavonoids*									
34	Rutin	0.84	1.09	4.81	610	609.1485	609 > 301 > 271, 179	C_27_H_29_O_16_	3.73
35	Quercetin-3-glycoside	1.47	1.09	4.95	464	463.0905	463 > 301 > 271, 179, 151	C_21_H_19_O_12_	5.012
36	Isorhamnetin 3-*O*-β-d-glucoside	0.57	1.61	5.23	478	477.1061	477 > 314 > 300, 285	C_22_H_21_O_12_	4.613
37	Casticin	1.44	1.15	7.42	374	373.0937	375 > 358 > 343 > 328	C_19_H_17_O_8_	2.142
*Etc*									
38	Shikimic acid	1.17	1.34	1.31	174	173.0463	173 > 155, 129	C_7_H_9_O_5_	4.238
39	Pantothenic acid	1.37	1.21	1.46	219	218.1044	218 > 187 > 143, 130	C_9_H_16_O_5_N	4.604
*N.I*									
40	N.I 1	1.51	1.08	6.63	174	173.1190	173 > 127 > 123, 97	-	-
41	N.I 2	1.50	1.06	6.86	926	925.4467	939 > 808 > 645	-	-
42	N.I 3	1.51	1.07	6.90	216	215.1298	215 > 173 > 127	-	-

^a^ VIP: variable in projection; ^b^ RT: retention time; ^c^ mass to charge ratio for [M − H]^−^.

**Table 3:** Blood chemistry in control and HFD-fed C57BL/6 rats treated with AW and GW.

Group			Blood Chemistry						
			AST (U/L)	ALT (U/L)	GLU (mg/dL)	TG (mg/dL)	T-Chol (mg/dL)	HDL-C (mg/dL)	LDL-C (mg/dL)
ND	N	Mean	94.00	29.38 *	118.00 **	74.00 **	113.13 **	81.88 **	9.63 **
	8	SE	8.80	1.05	10.49	3.56	5.11	1.14	0.26
HFD Control	N	Mean	122.75	76.75	226.00	97.75	210.75	97.13	18.88
	8	SE	5.57	8.07	4.76	5.31	8.39	2.73	1.33
AW 100	N	Mean	163.38 *	122.50 *	212.63	88.13	195.75	96.50	20.00
	8	SE	20.60	31.01	12.40	2.40	7.57	2.28	1.46
AW 300	N	Mean	96.63	67.00	210.50	101.13	177.50 **	89.25 *	16.13
	8	SE	7.25	8.61	17.02	7.17	9.60	2.94	1.42
GW 100	N	Mean	117.63	58.88	192.25 *	92.13	182.13 **	95.88	14.75 *
	8	SE	12.06	7.49	5.58	1.54	5.40	2.84	0.70
GW 300	N	Mean	137.88	46.63	203.13	80.00 **	163.25 **	91.50	13.63 **
	8	SE	8.01	3.30	7.90	3.33	6.30	2.17	0.91

ND: Normal diet; HFD: 60% high-fat diet; AW 100: HFD + fermented annual wormwood (AW) extracts 100 mg/Kg; AW 300: HFD + fermented AW extracts 300 mg/Kg; GW 100: HFD + fermented glasswort (GW) extracts 100 mg/Kg; GW 300: HFD + fermented glasswort extracts 300 mg/Kg. Data are presented as mean ± SD. Significant difference from HFD using *t*-test: * *p* < 0.05, ** *p* < 0.01.

## Data Availability

The data presented in this research are available in this article.
